# Experimental heatwaves reduce the effectiveness of ejaculates at occupying female reproductive tracts in a model insect

**DOI:** 10.1098/rsos.231949

**Published:** 2024-05-08

**Authors:** Kris Sales, Paul Thomas, Matthew J. G. Gage, Ramakrishnan Vasudeva

**Affiliations:** ^1^ Forest Research, Inventory, Forecasting and Operational Support (IFOS), Farnham GU10 4LH, UK; ^2^ School of Biological Sciences, University of East Anglia, Norwich NR4 7TJ, UK; ^3^ School of Biology, University of Leeds, Leeds LS2 9JT, UK

**Keywords:** climate change, sperm behaviour, thermal stress, spermatheca

## Abstract

Globally, heatwaves have become more common with hazardous consequences on biological processes. Research using a model insect (*Tribolium castaneum*) found that 5-day experimental heatwave conditions damaged several aspects of male reproductive biology, while females remained unaffected. However, females’ reproductive fitness may still be impacted, as insects typically store sperm from multiple males in specialized organs for prolonged periods. Consequently, using males which produce sperm with green fluorescent protein (GFP)-tagged sperm nuclei, we visualized *in vivo* whether thermal stress affects the ejaculate occupancy across female storage sites under two scenarios; (i) increasing time since insemination and (ii) in the presence of defending competitor sperm. We reconfirmed that sperm from heatwave-exposed males sired fewer offspring with previously mated females and provided new scenarios for *in vivo* distributions of heat-stress-exposed males’ sperm. Sperm from heatwave-exposed males occupied a smaller area and were at lower densities across the females’ storage sites. Generally, sperm occupancy decreased with time since insemination, and sperm from the first male to mate dominated the long-term storage site. Reassuringly, although heated males’ ejaculate was less successful in occupying female tracts, they were not lost from female storage at a faster rate and were no worse than control males in their offensive ability to enter storage sites occupied by competitor sperm. Future work should consider the potential site-specificity of factors influencing sperm storage where amenable.

## 1. Introduction

Climate change is the greatest threat that biodiversity faces in the twenty-first century [1]. It is likely that by 2100 mean global temperatures will rise beyond 2°C relative to pre-industrial conditions [2] and associated with this warming is an escalation of climatic variability and extreme events [3]. The definition of heatwave varies but often incorporates a period of abnormally hot weather relative to a reference threshold, which can last from 2 days to months [4]. Heatwaves are predicted to continue increasing in frequency, duration and intensity [4,5]. Many studies have highlighted climate-induced biodiversity loss over decades [6–8], with extreme events potentially being more damaging than gradual mean temperature increases [9]. However, the biological mechanisms underlying such declines remain tentative [10,11].

A likely mechanism behind some climate-related extinctions is the heat liability of male reproductive biology. The knowledge of heat-stress-induced infertility in male endotherms was well established, primarily in endeavours to improve fertility in humans and domesticated animals [12]. For example, treating male mice (*Mus musculus*) scrota with 32°C air caused approximately 80% reductions in fertilization rates, relative to controls [13]. However, until recently, ectotherms’ potential for heat-induced infertility received little attention, despite their abundance [14], ecological importance [15] and potential vulnerability [16].

An emerging body of research in ectotherms shows that male reproduction is compromised by environmentally relevant sub-lethal heat-stress temperatures [17–21]. Moreover, inter-species comparisons conclude that male ‘thermal fertility limits’, the temperature range where males can reproduce, rather than viability limits, the temperature range where individuals can survive, determine population distributions and adaptive capacities [22–24]. Consequently, there is support for deepening and widening our understanding of the interaction between temperature extremes and fertility [25,26].

Often, studies on ectotherms find that heat stress damages the offspring production of males, without investigating the underlying causes that could be interlinked. Heat stress can compromise male fertility by (i) impacting on ejaculate traits important for entering and navigating the female tract to achieve fertilizations [27–29], (ii) damaging the genetic integrity of sperm and subsequent offspring development [20,30], and/or (iii) reducing the effectiveness of mating behaviour [31–33].

Previously, we explored the impacts of heatwaves on male reproduction using the red flour beetle (*Tribolium castaneum*) [33–35], a tractable insect model to investigate reproduction [36] and thermal stress [37]. We followed a common heatwave definition of 5-day periods when local daily thermal maxima surpass the long-term average maximum by 5°C [38]. Using 5-day experimental heatwave conditions at temperatures 5–7°C greater than the optimum for population productivity [37], we found that the reproductive fitness of heated males, and mature sperm numbers stored inside females, was half that of experimental controls. We linked low offspring production to reductions in mating frequency, testes volume, ejaculate sperm counts, sperm viability, sperm presence in the female tract, male-induced fecundity, ovum hatchability and transgenerational offspring fitness [34].

Our recent work showed that heat stress lowered male fertility and perturbed subsequent sperm presence in the female tracts [34]; visualized *in vivo* by using a transgenic stock with males expressing green fluorescent protein (GFP)-tagged sperm heads. However, this initial research on sperm storage dynamics was limited, being a single coarse measure and representing a relatively rare and restricted scenario of a single timepoint following a virgin mating. First, female insects store sperm in specialized organs prior to fertilization [39], sometimes for weeks in the absence of remating [40–42]. Second, these organs vary across species, are often complex, and contain two important sites: the bursa copulatrix and the spermatheca, which some regard as short-term and long-term storage sites, respectively [36,43]. Third, polyandry is widespread [44,45], and, with sperm storage, sperm competition is the normal route to reproductive success for many insects [46]. Consequently, as only subsets of ejaculates are likely to be held in specific areas of storage organs, for prolonged periods and in the presence of competitors prior to fertilization, these dynamics should be considered but have been relatively unexplored in thermal research.

*Tribolium castaneum* is a suitable modecg promiscuous with high remating rates and complex sperm storage structures. Moreover, previous research incorporating competition [43,47] and temporal dynamics [40] has linked female-stored sperm abundance with reproductive success. Here, we provide greater resolution of the interaction between heat-stress exposure, time elapsed since mating and competition on sperm across the two female storage sites: (i) bursa and (ii) spermatheca. Furthermore, rather than relying on just one metric of sperm occupancy, we assayed multiple quantifications of sperm density and spread. We hypothesized that heat-stress-exposed sperm would significantly (i) reduce the rate at which sperm migrated into the spermatheca, (ii) increase the rate at which sperm depleted within the bursa, and (iii) reduce the ability to displace competitor sperm.

## 2. Material and methods

### 2.1. *Tribolium castaneum* strains and maintenance

Stocks were maintained in standard conditions of a 16L:8D-photoperiod, 30 ± 0.5°C, 60 ± 5% RH and ad libitum fodder. Fodder consisted of plain white organic flour (Dove’s Farm Foods Ltd, Berkshire) and powdered organic brewer’s yeast (9:1 by volume; ACROS organics, Belgium), topped with organic oats (Morning Foods Ltd, Cheshire) [31]. Populations were cultured as non-overlapping generations in 1 l pots, renewed every 35 days upon life cycle completion. On generation renewal, approximately 300 sexually mature adults were transferred to fresh fodder for 7 days to reproduce, before their removal with an 850 μm manual mesh sieve (Endecotts Ltd, London, UK).

**Figure 1.**
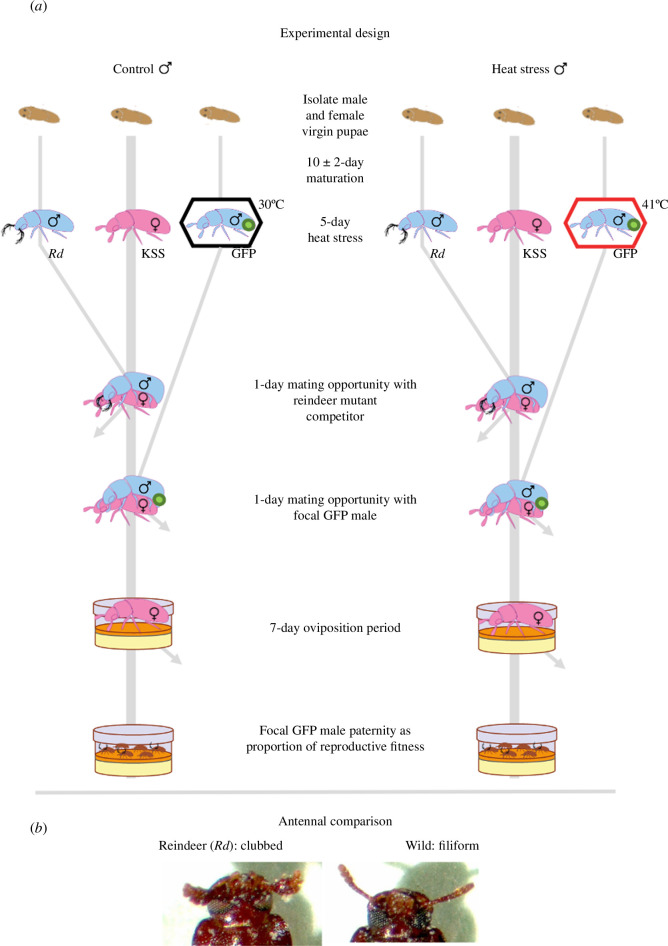
Protocols to assess the competitiveness of sperm (**P_2_**) after exposure to heat-stress conditions. (*a*) Each female was given an opportunity to mate with a competitor male carrying a homozygous dominant ‘*Reindeer*’ (*Rd*) mutation for clubbed antennae, which is inherited by all offspring sired. Each female was then provided with either a heat-stress-treated or control male carrying wild-type filiform antennae (and GFP-tagged sperm). The reproductive success of each male over the 7-day oviposition period was calculated by scoring the relative proportion of offspring with filiform (=GFP) or clubbed (=*Rd*) antennae produced by their common female mate. (*b*) Photo comparison of *Rd* and wild-type antennae.

Males were mainly from a transgenic stock bearing GFP tagged to protamine in their sperm heads. The stock was supplied in 2013 (Pitnick Laboratory, The Centre for Reproductive Evolution, Syracuse University); for details on its creation, see Droge-Young *et al*. [43]. GFP-expressing sperm enabled the *in vivo* visualization of sperm through the translucent walls of the female reproductive tract. In the experiment where the reproductive fitness during sperm competition was assessed, paternity was distinguished by antennal shape. Here, competitor males were from a transgenic stock, obtained from the Beeman Laboratory (Manhattan, Kansas) in 2010, which contained a dominant ‘*Reindeer*’ (*Rd*) mutation, maintained in a homozygous state [48]. Consequently, offspring sired by GFP males had wild-type filiform antennae while all offspring from *Rd* males inherited swollen clubbed antennae (figure 1). Stock individuals were ‘Kraków Super Strain’ (KSS), a genetically diverse population created in 2008 by combining 35–60 individuals from 11 different strains [49]. All the females and the competitor males used in the sperm distribution experiment were KSS. Prior to tests, we knew that 94% of *Rd* males (*n* = 34), 95% of GFP males (*n* = 92) and 100% of KSS males (*n* = 36) would inseminate females when provided with a 24 h mating opportunity.

### 2.2. Thermal exposure

Heat stress was informed by common heatwave definitions [38] and recent work using the KSS [34]. Treatments were set to a 5-day 41 ± 0.5°C stress, or 30 ± 0.5°C control conditions, at 60 ± 5% RH. The 41°C set-point was selected because (i) it is 5–6°C greater than the critical thermal optimum for several *T. castaneum* life-history traits [34,37], (ii) it is a geographically widespread summer heatwave temperature [50], and (iii) it does not cause excessive mortality in the GFP stock (and we observed 100% mortality at 42°C). There is a 20-year precedence in our research group to maintain stocks at 30°C because of logistical constraints within the controlled environment facility; being a few degrees below the optimum (figure 1) [34]. Thus, the heatwave-control effect sizes may be conservative.

Individuals were sexed based on genital dimorphism during the pupal stages[51], then stored in standard 30°C conditions for 10 ± 2 days after eclosion, to develop into sexually mature adults [52]. To limit pre-assay mating activity and sperm expenditure, males were kept singly in perforated Eppendorf tubes containing 0.5 g of fodder [31].

These sexually mature adults (10 ± 2 days old) were randomly allocated to standard 30°C control conditions or heatwave simulations. For heatwaves, Eppendorf racks were randomly stratified down the centre of Octagon 20 Eco incubators (Brinsea Incubation Specialists, Somerset), set to 41°C for 5 days. Randomization of position, daily rotations and periodic monitoring of temperature using calibrated mercury thermometers (G.H. Zeal, London) limited potential microclimatic confounds in the incubators. Following 5-day exposures, males were held for 24 h at 30°C, to ensure that post-heatwave quiescence had passed before experimental mating treatments. Prior to mate pairings, virgin 10 ± 2-day post-eclosion KSS females were marked with a small dot of correction fluid on the dorsal thorax [53], so they could be easily distinguished from males without impacting on reproduction; for further details, see Sales [49].

### 2.3. Sperm competition assay

The effect of heat stress on GFP male competitiveness was investigated by comparing the ability of heat-treated and control males in achieving insemination–fertilization success within KSS females, which had previously been mated to *Rd* males (P_2_) (figure 1). Logistics constrained running assays with P_1_ and P_2_; P_2_ was prioritized because it seems to have greater implications for males’ reproductive fitness, with last male paternity precedence and frequent remating being common in *T. castaneum* [34,36,47,54]. The halving of male reproductive competitiveness had been previously demonstrated using the KSS strain [34], but repeating the assay with GFP males: (i) reconfirmed the generality of male reproductive competitiveness being heat liable across populations and (ii) linked sperm distribution reductions observed *in vivo* more directly with reduced competitive offspring production.

During the GFP males’ post-heatwave treatment recovery day (see §3.2), isolated virgin KSS females were paired with virgin *Rd* males for a 24 h mating opportunity in 7 ml vials containing 0.5 g of fodder and held in standard 30°C conditions. After the 24 h mating opportunity, each *Rd* male was replaced with a virgin GFP male from the control (*n* = 19) or heatwave (*n* = 18) treatment. Following this second 24 h mate pairing, each GFP male was removed, and females were transferred to oviposit individually in 5 cm Petri dishes, containing 10 g of fodder, for 7 days. Dishes were maintained in 30°C conditions for 35 days for offspring development, until being frozen at −6°C. As *Rd* males produce offspring with clubbed antennae [48], relative paternity was assigned to the GFP male or *Rd* competitor, by scoring filiform or clubbed antennae, respectively. The second-male mating opportunity being 24 h was consistent with previous publications (e.g. [34]). However, we note a caveat that GFP males in this assay were likely to have had more remating opportunities than those given an hour in the sperm visualization assay. Consequently, GFP males would probably have transferred more sperm in the competition assay than in the visualization assay, which limits the inter-assay comparability.

### 2.4. Sperm distribution sample preparation

**Figure 2.**
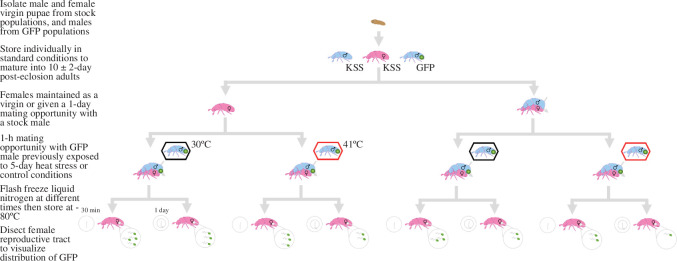
Protocols to elucidate factors influencing female sperm storage. Mature adult females were either kept as virgins or given a 24 h mating opportunity with stock males. Meanwhile, focal males expressing GFP-tagged sperm were exposed to 5 day heat-stress or control conditions. GFP males were then paired with the females for 24 h. Females were flash frozen in liquid nitrogen at two time points: 30 min or 24 h after the mating opportunity. Flash frozen females were stored at −80°C to preserve the integrity of the sperm distributions before dissections.

The factors affecting the sperm storage dynamics within the female reproductive tract were assessed using GFP males, KSS competitor males and KSS females (figure 2). GFP males were either exposed to a control or a heatwave treatment (see §3.2). During the GFP males’ 24 h recovery, half of the KSS females remained isolated as virgins while the others were given monogamous mating opportunities with virgin KSS males in vials for 24 h. The split treatment of the females generated the subsequent contrast between competitive and non-competitive sperm dynamic scenarios. Virgin GFP males were paired with either a virgin, or a previously mated, female for 1 h. This 1 h window ensured that all pairs mated, while limiting mating frequency variation between GFP males.

Males were removed and females flash-frozen with liquid nitrogen, either 30 min or 24 h later, to explore temporal dynamics of sperm distribution [43]. Therefore, treatments included GFP sperm storage in the absence of sperm competition at 30 min; sampling (*n*_control_ = 23; *n*_heat_ = 25) and 24 h (*n*_control_ = 24; *n*_heat_ = 25), plus storage in the presence of competition at 30 min (*n*_control_ = 22; *n*_heat_ = 24) and 24 h (*n*_control_ = 23; *n*_heat_ = 24).

After freezing, females were stored at −80°C. Sampling of frozen females for dissection was stratified and randomized. Only a couple of individuals were drawn from −80°C to be held on ice at a time immediately prior to dissection, and for every eight females, each treatment was represented in a random order. Dissections were conducted at ×20 magnification on a Zeiss Discovery V12 stereomicroscope (Carl Zeiss, Jena, Germany) using fine-tipped (0.10 × 0.06 mm) dissecting forceps (Dumont, Switzerland). Females were placed ventral side up in Grace’s insect medium (Thermo Fisher, MA). The reproductive tract was extracted by extruding and then pulling the ovipositor tip with forceps. Typically, this isolated the desired sperm storage section containing the larger bursa copulatrix and smaller spermatheca. Infrequently, some gastrointestinal tract and/or ovary tissue were also extracted; these sections were excised. The intact spermatheca and bursa were transferred to 30 µl of fresh buffer, then sealed under a 20 × 20 mm coverslip using impermeable instant contact adhesive (EVO-STIK, UK). The tract orientation and coverslip application method were kept constant across samples.

### 2.5. Sperm distribution image acquisition

Bright-field and fluorescence images of the whole tract were visualized with a Zeiss ×10, 0.3 NA Plan-Neofluar objective on an AxioPlan 2ie microscope, and images were captured using an Axiocam HRm CCD camera and Axiovision (v. 4.8.2) software. Greater resolution of the smaller spermatheca was achieved using a Zeiss ×20, 0.6 NA Plan-Apochromat objective. Green fluorescence, primarily produced from GFP sperm, was excited using a 472 ± 15 nm filter, and images were collected through a 520 ± 18 nm filter [55]. General red auto-fluorescence was excited through a 562 ± 20 nm filter, and images were collected using a 624 ± 20 nm filter. The channel-specific exposure times were kept constant for all images within regions. In particular, the bright-field, general auto-fluorescence and GFP channel exposure times were 5, 100 and 150 ms for the total reproductive tract and bursal images, but were 10, 50 and 75 ms, for the spermathecal images.

**Figure 3.**
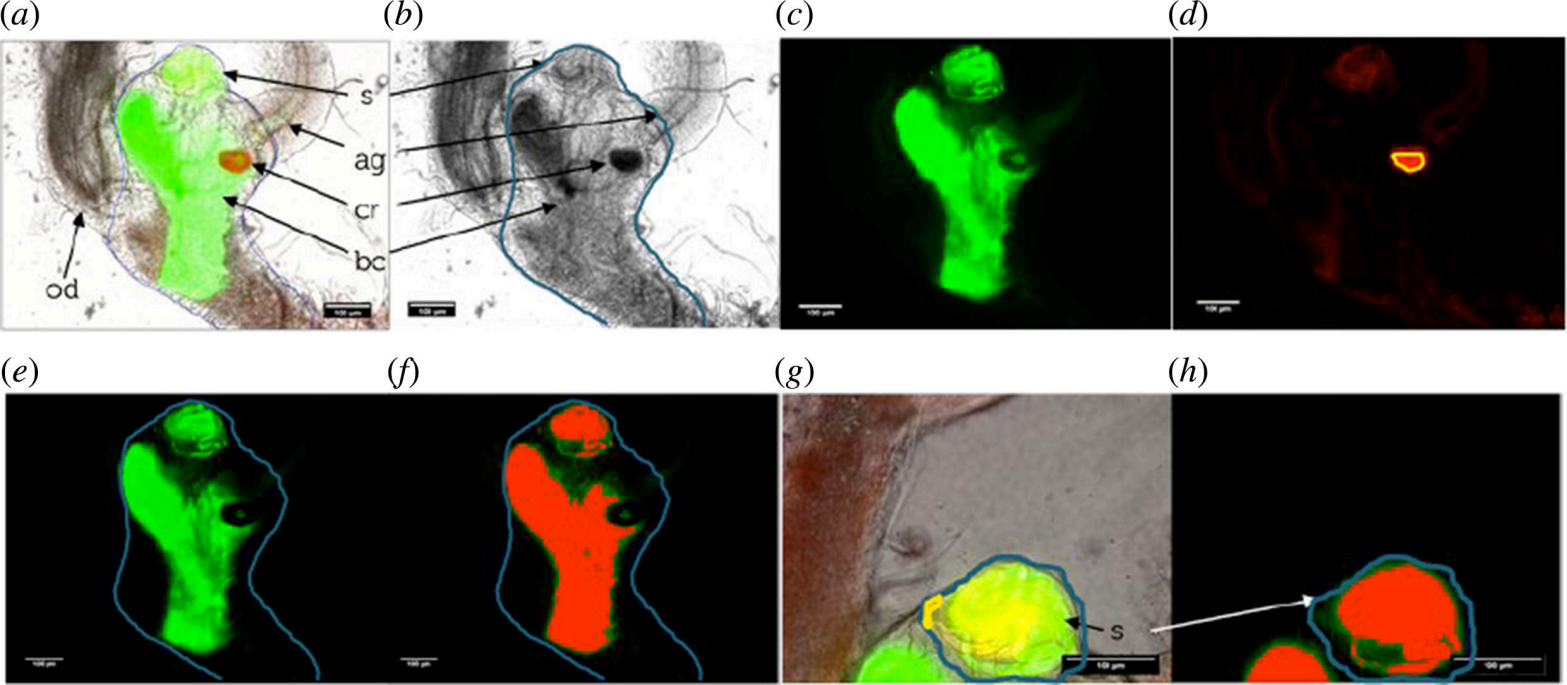
Acquisition and processing for sperm distribution assays. Female reproductive tract containing sperm whose heads are tagged with GFP. The labelled structures are bc, bursa copulatrix; s ,spermatheca; cr, chitin ring; ag, accessory gland and od, oviduct. Images (*a–f*) make up the total reproductive tract. (*a*) Fluorescence within the composite images of green 520 ± 18 nm, red 624 ± 20 nm and bright-field channels. (*b*) Region of interest (ROI) drawn (blue outline) around the walls of the reproductive tract using the bright-field image. (*c*) Fluorescence within the green channel, also consisting of general auto-fluorescence and the true GFP sperm heads. (*d*) Undesired auto-fluorescence captured in the red channel which is derived from structural features unrelated to the GFP sperm. A ROI (e.g. highlighted in yellow) is drawn inside areas showing clear auto-fluorescence in the absence of sperm. In total, the reproductive tract images (*a–f*); the chitin ring at the base of the spermathecal duct was used. Images showing the spermatheca (*g,h*) and the chitinous walls of spermathecal tubules were selected (as in (*d*)). The mean fluorescence intensity in this ROI was measured in both (*c*) (Int_GFP_) and (*d*) (Int_AF_) to calculate GFP sperm distribution. (*e*) Applying background subtraction to (*d*), which was then multiplied by the correction factor, Int_GFP_ divided by Int_AF_, to the resulting image (subtracted from (*c*)), leaving only GFP-sperm-derived fluorescence, quantifying the distribution. The mean intensity of this true sperm-GFP fluorescence was measured within the boundary of the ROI (blue region outlined) using the bright-field image (*b*).**(*f*** )The ‘intermodes’ threshold algorithm was used here, this created a binary image with pixel values. Those pixel values above the threshold being classed as fluorescent (highlighted in solid red, (*f*)). Pixel values below the threshold are grouped as ‘non-fluorescent’. The percentage cover of the fluorescent area within the bright-field ROI (blue outline) was calculated (*b*). Images (*g,h*) of the spermatheca (s). Composite image of bright-field channel (*g*) shows the red and green fluorescence with an auto-fluorescence within this ROI. The output of a green fluorescence channel (*h*) shows the image which has undergone background subtraction to correct for auto-fluorescence. This was done by drawing a ROI around the outer wall of the spermathecal tubule which was used to calculate a mean fluorescence value. This ROI calculated the fluorescence percentage cover after image thresholding.

Axiovision images (14-bit greyscale) were analysed using a Fiji (ImageJ, v. 1.49k) custom-written macro which focused on isolating and quantifying the signal (GFP-sperm fluorescence) from the noise (auto-fluorescence) [56]. The process of background subtraction, auto-fluorescence correction, region of interest definition, thresholding and sperm dynamic metrics are described for the total tract (figure 3*a*–*f*) and the spermatheca (figure 3*g,h*). The macro minimized fluorescence background noise and uneven illumination from the female tract, using *background subtraction* with a *rolling ball* radius of 25 pixels for the smoothing algorithm (figure 3 [57]). To remove false GFP auto-fluorescence caused by chitinous structures like the accessory gland ring and ovipositor, so that only true GFP sperm head fluorescence was visible, an *auto-fluorescence correction* macro was applied specific to each sample [58]. The macro corrected the GFP-channel image by manually drawing a *region of interest* (ROI) in the auto-fluorescence channel on an area of the image displaying high general red fluorescence, but minimal sperm-derived GFP fluorescence. The high auto-fluorescence structure used was the chitin ring at the base of the spermathecal duct for the total reproductive tract, and the outer tubule walls for spermathecal images. The mean intensity was measured in this ROI (Int_Auto_). The same ROI was then also applied to the GFP-channel image to measure the mean intensity (Int_GFP_). A correction factor was determined by dividing Int_GFP_ by Int_Auto_. The auto-fluorescence channel image (figure 3*d*) was multiplied by the correction factor, then the auto-fluorescence-corrected image was subtracted from the GFP-channel image (figure 3*c*), leaving primarily GFP sperm-derived fluorescence for measurement (figure 3*e*). The total tract and spermatheca storage area ROIs were defined by tracing their perimeter walls in the bright-field image [59] (figure 3*b*).

For each sample, multiple fluorescence measures were investigated to score sperm occupancy. First, observable sperm presence or absence was recorded for the spermatheca and total tract. Second, mean and median pixel intensities were determined for the total tract and the spermatheca ROIs, in 16-bit greyscale. Finally, the percentage cover of fluorescence within the ROIs was calculated by applying a *binary threshold* to the image, which converted pixels to zero or one if they registered as not fluorescent or fluorescent, respectively (figure 3*f*) [59]. For quantifying fluorescence distribution within Fiji, ‘*intermodes*’ was selected, which maximized the detection of true GFP fluorescence while limiting the inclusion of false auto-fluorescence [60]. The fluorescence percentage cover within the respective ROIs of the total tract or spermatheca was derived from the fluorescent pixel frequency, divided by the total pixel number, multiplied by 100.

This macro method has been published previously [34,49]. Although efficient, it is currently unable to produce precise sperm quantification, but using fluorescence as a proxy of cell abundance has been highlighted in research on human sperm [61] and bacterial culture [62]. Qualitatively, the method produced negligible fluorescence values for reproductive tracts from females which are virgin or have only mated non-GFP males. Tracts with high GFP values had abundant sperm apparent when dissected. The method has been consistently using inter-researcher repeatability analysis; e.g. the mean *R* ^2^ = 0.97 ± 0.01 s.d. for three researchers measuring mean GFP intensity on a sample of 200 images. Furthermore, heatwaves were very unlikely to have affected GFP fluorescence as it is stable at temperatures above 60°C [63,64]. Finally, the results observed from this GFP sperm visualization methodology are comparable with another independent study quantifying GFP sperm with manual counts [43].

### 2.6. Statistical analysis

For data analysis, we used RStudio.2022.07.2+576 in R v. 4.2.1 [65]. Boxplots were created in ‘ggplot{ggplot2}’ [66], and diagrams in Adobe Illustrator (Adobe Systems, San Jose, CA) and PowerPoint (Microsoft, Redmond, WA). All test statistics are recorded to one decimal place, and all *p*-values to three decimal places. Descriptive statistics (mean ± s.e.) were calculated using ‘describeBy{psych}’ [67]. Table 1 lists brief model summaries for each experiment including the overall significance of its fixed factors, error distribution and link function, sample size, explained variance and random factors where applicable. Electronic supplementary material, table S1 incorporates table 1 along with parameter-level-specific information comprising descriptive statistics, beta estimates, *z*/*t* scores and *p*-values. Sample sizes for sufficient statistical power were informed by recent work [34].

**Table 1.** Brief summaries for models investigating the effects of heat-stress exposure, time since insemination and male competition status on sperm distribution in the *T. castaneum* female. Descriptive statistics and factor-level betas, *t*/*z*-statistics and *p*-values are in electronic supplementary material, table S1.

experiment	fixed factor	d.f.	*χ*2 / *F*	*p*	model, error distribution (and link function)	*R* ^2^*
GFP male P_2_ paternity proportion	male thermal treatment	1	*χ* ^2^ = 4.6	0.031	GLM Q-bin (logit)	10%
	residual	35				
female tract sperm distribution mean intensity	male thermal treatment	1	*F* = 16.0	<0.001	GLM Gaus (ID)	49%
	competitor presence	1	*F* = 0.3	0.603		
	time	1	*F* = 159.4	<0.001		
	residual	187				
female tract sperm distribution percentage cover	male thermal treatment	1	*χ* ^2^ = 26.1	<0.001	GLM Q-bin (logit)	46%
	competitor presence	1	*χ* ^2^ = 0.4	0.518		
	time	1	*χ* ^2^ = 157.3	<0.001		
	residual	187				
female spermatheca sperm distribution mean intensity	male thermal treatment	1	*F* = 36.9	<0.001	GLM Gaus (ID)	41%
	competitor presence	1	*F* = 93.0	<0.001		
	time	1	*F* = 0.5	0.483		
	residual	187				
female spermatheca sperm distribution percentage cover	male thermal treatment	1	*χ* ^2^ = 12.6	<0.001	GLM Q-bin (logit)	31%
	competitor presence	1	*χ* ^2^ = 69.8	<0.001		
	time	1	*χ* ^2^ = 1.5	0.220		
	residual	187				

*[68]

All data were analysed with generalized linear models (GLMs) in ‘glm{stats}’ [69]. Diagnostic residual plots were examined using ‘plot{graphics}’ [70], and overdispersion, excessive variance: mean, assessed with a function [68; p. 110], The diagnostic plots and overdispersion metrics informed selection of the most appropriate error distribution for each GLM [68,71,72].

Initial maximal models were initially fitted with all relevant fixed factors. Three-way interactions were excluded from initial models owing to conceptualization difficulties [68]. Two-way interactions were entered initially but then dropped if they were not statistically significant during progress towards minimum adequate models [71]. Fixed factors were retained regardless of *p*-value as they were conceptually important to experimental design [73]. The importance of the experimental treatment variables, and their two-way interactions, was measured using Akaike’s information criterion (AIC) comparisons, and log-likelihood ratio tests (LLRTs) with and without the term of interest using ‘drop1{stats}’ [68,69]. For LLRTs, we used *F* tests when the response variable was continuous, and *χ^2^* tests when it was a bounded proportion [68]. After assessing overall interaction and factor significances, simple *post hoc* comparisons producing *z* statistics and *p*-values between treatment groups and controls were derived from ‘summary(model){stats}’ [71]. Pseudo *R* ^2^ (explained deviance) was calculated to indicate how much variation in the response variable was captured by the model [68].

The effect of GFP male heat-stress exposure on the insemination–fertilization competitiveness of their sperm was initially fitted with a logit-linked binomial GLM. However, the error distribution was changed to quasi-binomial to account for overdispersion [68]. GFP paternity was a proportion entered as a paired variable with ‘cbind(success, fail){base}’, where success was the frequency of offspring with filiform antennae sired by the GFP male, and fail was the number with clubbed antennae sired by the *Rd* competitor [68]. The GFP male heat treatment (control or heat) was entered as a fixed factor.

For the sperm distribution analysis, all GLMs contained three two-level categorical fixed factors. First, the GFP male treatment (control or heat) before mating. Second, the female mating status prior to the GFP male (virgin or KSS mated). Third, the time delay (30 min or 24 h) between male GFP male removal and female flash-freezing. Two-way interactions between the fixed factors were input, but were not statistically significant, so were excluded for parsimony [71].

Four GFP sperm distribution measures were recorded: median pixel intensity, mean pixel intensity, percentage cover and presence. However, these variables were correlated (electronic supplementary material, figure S1). Presence and percentage cover were related in the total tract (*S*_(190)_ = 527 700, *p* < 0.001, *R*_s_ = 0.54), and the spermatheca (*S*_(190)_ = 438 390, *p* < 0.001, *R*_s_ = 0.62). Similarly, median and mean intensity were linked in the total tract (*S*_(190)_ = 93 241, *p* < 0.001, *R*_s_ = 0.92), and the spermatheca (*S*_(190)_ = 65 823, *p* < 0.001, *R*_s_ = 0.94). Owing to strong correlations and similar responses across treatments [68], only full analyses of the mean intensity and percentage cover are included in the results. Models of sperm presence and median intensity are reported in electronic supplementary material, table S1. Mean intensity and percentage cover are not perfectly correlated and capture slightly different ejaculate metrics; percentage cover indicates the extent of the spread of the sperm across the reproductive tract while mean fluorescence intensity indicates the sperm density.

The percentage cover of sperm in the total female reproductive tract, and in the spermatheca, produced overdispersed proportion data bound between zero and one. Therefore, both were analysed with logit-linked quasi-binomial GLMs. The response variable was paired using ‘cbind(success, fail){base}’, where success was the percentage of the ROI displaying GFP fluorescence and failure was the percentage lacking it. Both the mean GFP fluorescence pixel intensity in the total tract, and in the spermatheca, produced unbound continuous data with values ranging between 11277.0 and 6.3. Therefore, data were fitted with identity-linked Gaussian GLMs.

## 3. Results

### 3.1. Impact of male heat-stress exposure on reproductive fitness of sperm in competition

**Figure 4.**
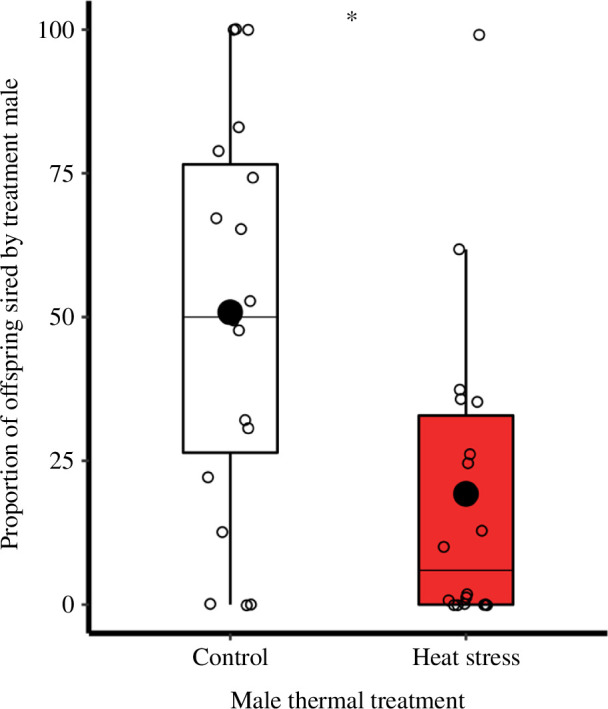
Impact of heat stress on the sperm competitiveness (**P_2_**) of treatment males, indicated by proportion of offspring they sire with previously mated females. Relative success of control (*n* = 19) and heat-treated (*n* = 18) males, whose sperm express GFP, in gaining paternity with a female previously mated to a rival control male (**P_2_**). Boxplots display a median line, interquartile range (IQR) boxes, 1.5 × IQR whiskers, a mean dot and data points. Significance thresholds: *p < 0.050.

Sperm produced from GFP males exposed to 41°C for 5 days were less able to achieve fertilization success in females already mated to control males (χ^2^_(1,35)_ = 4.6, *p* = 0.031, *n*_30°C_ = 19, *n*_42°C_ = 18; figure 4). In particular, the proportion of offspring a second mate (P_2_) sired over 7 days was 63% lower if they had previously been thermally stressed, relative to controls (*z* = −2.1, *p* = 0.043).

### 3.2. Sperm distribution in the total reproductive tract

**Figure 5.**
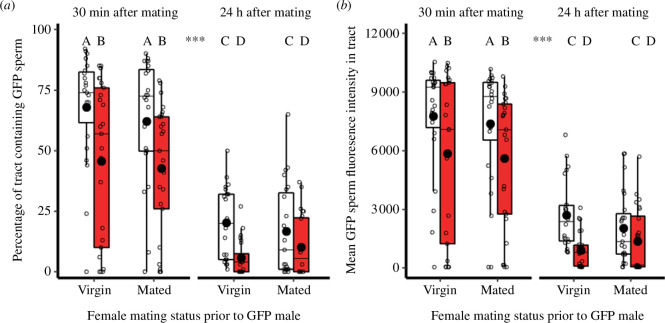
Effect of male heat-stress exposure on sperm competition and temporal dynamics on sperm distributions throughout the female reproductive tract. Males expressing GFP-tagged sperm were treated with 5-day 41°C stress (red) or 30°C control (white) conditions prior to mating. Before being paired with GFP males, females were virgin or previously mated to stock males. Flash freezing and dissection occurred at two time-points. The total reproductive tract includes the bursa copulatrix and spermatheca. (*a*) GFP sperm spread. (*b*) GFP sperm density (mean pixel fluorescence intensity). For both figures, sample sizes from left to right (*n* = 23, 25, 24, 25, 22, 24, 23, 24). Boxplots display a median line, interquartile range (IQR) boxes, 1.5 × IQR whiskers, a mean dot and data points. Significance thresholds: ***p < 0.001, letters denote differences between treatments.

Sperm from heat-stressed GFP males were stored differently throughout the total female reproductive tract, compared with controls. Specifically, sperm from heat-treated males were often absent in parts of the bursa copulatrix including the central anterior area between the chitin ring and spermatheca, and the posterior section closest to the ovipositor. Accordingly, the percentage of the total tract occupied by sperm was affected by the male thermal treatment (χ*^2^*_(1,189)_ = 26.1, *p* < 0.001, *n*_30°C_ = 92, *n*_42°C_ = 98; figure 5*a*). Generally, sperm from heat-stressed males occupied 32% less tract area, relative to controls (*z* = −5.0, *p* < 0.001). Similarly, the average fluorescence intensity, indicative of sperm density in the tract, was altered by the male thermal treatment (*F*_(1,189)_ = 16.0, *p* < 0.001, *n*_30°C_ = 92, *n*_42°C_ = 98; figure 5*b*). On average, the mean sperm fluorescence from heat-treated males was 21% lower than controls (*t* = −4.0, *p* < 0.001).

Time since spermatophore transfer also influenced the area-cover spread of sperm in the female tract (*χ^2^*_(1,189)_ = 157.3, *p* < 0.001, *n*_30 min_ = 97, *n*_24 h_ = 93). Similarly, time affected the density of sperm in the tract (*F*_(1,189)_ = 159.4, *p* < 0.001, *n*_30 min_ = 97, *n*_24 h_ = 93). On average, the percentage cover of sperm was proportionally 72% lower after 24 h had elapsed, compared with 30 min (*z* = −11.3, *p* < 0.001). Furthermore, across both the heat-stress and control treatments the mean decrease in fluorescence intensity was 65% over 24 h (*t* = −12.6, *p* < 0.001). Conversely, the prior presence of stock competitor male sperm, inseminated over the preceding 24 h, did not alter the spread (*χ^2^*_(1,189)_ = 0.4, *p* = 0.518; *z* = −0.6, *p* = 0.518, *n*_virgin_ = 94, *n*_comp_ = 96), or density (*F*_(1,189)_ = 0.3, *p* = 0.603; *t* = −0.5, *p* = 0.603, *n*_virgin_ = 94, *n*_comp_ = 96), of GFP male sperm.

There were no significant interactions between the male thermal treatment and the other treatments measured; in particular, the rate of sperm loss from the female tract was not faster if the ejaculate came from a heat-stressed male, and likewise, ejaculates from heated males were not excessively worse at entering female tracts filled with defending competitor sperm.

### 3.3. Sperm distribution in the spermatheca

**Figure 6.**
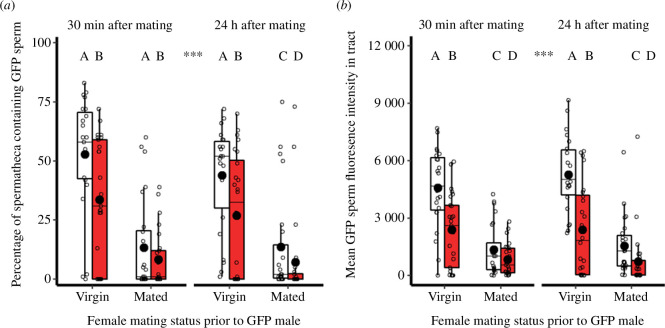
Effect of male heat-stress exposure on sperm competition and temporal dynamics on sperm distributions in the spermatheca. Males expressing GFP-tagged sperm were treated with 5-day 41°C stress (red) or 30°C control (white) conditions prior to mating. Before being paired with GFP males, females were virgin or previously mated to stock males. Flash freezing and dissection occurred at two time-points. (*a*) Spread of the area of GFP sperm as the percentage fluorescing above a threshold within the coverage area. (*b*) GFP sperm density as the mean pixel fluorescence intensity. For both figures, sample sizes from left to right (*n* = 23, 25, 24, 25, 22, 24, 23, 24). Boxplots display a median line, interquartile range (IQR) boxes, 1.5*IQR whiskers, a mean dot and data points. Significance thresholds: ***p < 0.001, letters denote differences between treatments.

Paralleling the pattern in the total reproductive tract, the male thermal treatment prior to mating affected the spread of the area-cover spread (*χ^2^*_(1,189)_ = 12.6, *p* < 0.001, *n*_30°C_ = 92, *n*_42°C_ = 98; figure 6*a*) and density (*F*_(1,189)_ = 36.9, *p* < 0.001, *n*_30°C_ = 92, *n*_42°C_ = 98; figure 6*b*) of sperm stored in the spermatheca. Specifically, sperm from heat-stress exposed males occupied 34% less spermathecal area than controls (*z* = −3.5, *p* < 0.001), and displayed a mean 36% reduction in sperm density across treatments (*t* = −6.1, *p* < 0.001).

The spread (*χ^2^*_(1,189)_ = 69.8, *p* < 0.001, *n*_virgin_ = 94, *n*_comp_ = 96) and density (*F*_(1,189)_ = 93.0, *p* < 0.001, *n*_virgin_ = 94, *n*_comp_ = 96) of GFP male sperm was primarily influenced by whether the spermatheca was occupied with stock competitor sperm inseminated in the preceding 24 h. Across thermal treatments, the percentage cover of GFP sperm in the spermatheca was 70% smaller in females that had previously mated to competitor males, compared with virgins (*z* = −7.7, *p* < 0.001). Similarly, the mean spermathecal sperm density of control and 41°C-exposed GFP males was 58% lower in previously mated females (*t* = −9.6, *p* < 0.001). Conversely, time since spermatophore transfer had no impact on the spread (*χ^2^*_(1,189)_ = 1.5, *p* = 0.220, *n*_30 min_ = 97, *n*_24 h_ = 93; *z* = −1.2, *p* = 0.218) or density (*F*_(1,189)_ = 0.5, *p* = 0.483, *n*_30 min_ = 97, *n*_24 h_ = 93; *t* = 0.7, *p* = 0.483) of GFP sperm in the spermatheca.

Significant interactions between the male thermal treatment and the other factors measured were absent for the spermatheca. Thus, sperm from heat-stressed males were not worse at entering a spermatheca containing competitor sperm. Furthermore, the temporal dynamics of GFP sperm in the spermatheca did not change if they had come from a heat-treated male.

## 4. Discussion

We found that ejaculates from heat-stressed males: (i) sired fewer offspring in the presence of competitor sperm, (ii) were less dense and widespread across the female reproductive tract, and (iii) were less dense and widespread within the spermatheca. Despite general declines in sperm occupancy with both time passed since mating, and the prior presence of competitor sperm, we did not find that sperm produced from heat-exposed males were (iv) lost from the tract faster or (v) especially worse at displacing competitor sperm.

Heat-stressed males produced sperm which was approximately 33% less successful in occupying the bursa and the spermatheca, on average. In the bursa, losses primarily arose in the anterior distal section by the spermatheca, and in the posterior section near the oviduct. In *Tribolium*, female storage dynamics seems little explored with regard to temperature-stressed sperm (34). However, some research exists for the effect of other stressors applied to males on the dynamics of ejaculate storage in females. For example, *T. castaneum* males fed low-quality diets produced ejaculates which were 30% less likely to be inseminated and 10% less likely to be correctly positioned in the anterior bursa, which resulted in 40% fewer sperm in the whole female tract and 60% less sperm in the spermatheca [74]. Outside *Tribolium* some research has explored the impact of heat stress on subsequent sperm storage in females [27,75,76]. For example, *Drosophila virilis* females were unable to protect stored sperm when exposed to temperatures lower than those that damage mature sperm in males [77]. Moreover, in the springtail (*Orchesella cincta*), spermatophores of heat-exposed males exposed to 37°C were 25% less likely to be received by females, and half as likely to produce offspring than 20°C controls [78].

A 41°C exposure could have damaged several aspects of the ejaculate, explaining its impaired storage (figures 4 and 5), likelihood of contacting ova [79,80] and subsequent probability of producing offspring (figure 3). Reduced sperm occupancy was probably related to less being initially transferred. Across the limited range of research on ectotherms, lowered counts seem the most commonly reported form of sperm damage [27,78,81,82]; in *T. castaneum,* we have previously found 75% fewer ejaculated sperm after heat stress [34]. Depressed sperm counts could be driven by a reduction in testis volumes [27,34,83] and male copulatory performance [84,85], as reported in *T. castaneum* and other organisms. The poor occupancy of heated ejaculates could be owing to damage affecting sperm traits necessary for their migration and persistence in the female reproductive tract. First, spermatophores from heated *T. castaneum* males appeared disorganized and degraded [34], which may have hindered correct eversion and sperm dispersion [86]. Second, in *T. castaneum* [34] and other species [29,87], heat stress kills a portion of sperm, rendering them unable to actively disperse. High temperatures can impact on sperm viability both while they are in male or in female storage [29]. Third, exposure to higher temperatures may have partially reduced movement by affecting flagellar activity [27,88] and morphology [28]. Fourth, heat stress could denature and/or alter the expression of seminal fluid proteins, known to influence female behaviour related to sperm use [89–91,92]. Generally, the failure of heat-exposed male sperm to occupy the correct anterior bursal and spermathecal storage sites could render a greater proportion vulnerable to displacement by muscular contractions, ova migration and male intromissions [43,74,86]. Although the visualization assay highlights the abundance of sperm present, it is probably an upper estimate of male fertility as it does not quantify sperm quality. Sperm that may be on the verge of dying or have compromised genetic integrity rendering them non-viable can pose increased risk of subsequent embryonic failure or producing poor quality offspring as a consequence (e.g. [93,94]). Heat-linked damage mechanisms have been documented in several mammals and *Drosophila* species using *in vivo* and *in vitr*o assays (reviewed in [94,95]) and this includes oxidative stress[96], abnormal morphology [93], nucleotide breaks [97], altered methylation [98] and disorganized chromatin packing [93].

Time since spermatophore transfer, on average, reduced bursal sperm volume by two-thirds over 24 h. However, it had no effect on sperm volume presence in the spermatheca. In *T. castaneum* bursal sperm counts decrease with time since insemination [40]. For example, a manual sperm count study with the same GFP strain, and at similar time points, also found that bursal sperm declined by a third over 24 h [43]. Authors attribute significant bursal sperm loss to spermatophore ejection [40] and oviposition [43], a couple of hours after mating. We found sperm in the spermatheca 30 min after mating, in line with reports that the migration there starts 10 min after insemination [40]. In this study, the spermathecal sperm occupancy was constant over 24 h. However, others report an increase in sperm migration over the first 2 h in unmated [40,99] and previously mated [43] *T. castaneum* females.

Our reproductive fitness assay conformed to the common phenomenon of last mate precedence in *T. castaneum* [74,99], perhaps explained by its limited post-copulatory mate guarding, poor sperm retention and lack of mating plugs [40,43]. Conversely, success of the most recently inseminated sperm at occupying the spermatheca decreased by two-thirds if competitor sperm were already defending it. Other *T. castaneum* research finds that sperm defending the long-term storage site are relatively difficult to displace [43,54], in contrast to other species like *Drosophila melanogaster* [100,101]. The relative difficulty of sperm displacement in the *T. castaneum* spermatheca could arise from sperm stratification in the elongate storage tubules over the first hour of deposition [99,102,103], tubules being too narrow for aedeagi entry to remove sperm [104,105] and/or the area being relatively distal from muscular and ova movements [74].

The sperm from both temperature exposures performed similarly across time and prior female mating status treatments. This result was unexpected, because we predicted that as female reproductive tracts tend to be mechanically and chemically hostile environments [74,106], they could compound prior heat stress, and cause a greater decline in performance. The lack of synergistic interactions between heat stress, sperm competition and time suggests there may be a subset of sperm successful at entering, and persisting in, the tract which is relatively unaffected by previous heat stress. Alternatively, it may suggest sperm are comparatively resistant to multi-stressor environments. An initial step to addressing such hypotheses would be to repeat the assay with larger sample sizes and confirm non-significant interactions were not owing to type 2 error.

We discovered that sperm from heated males were less successful at being stored inside females. However, the study cannot easily elucidate the relative importance of male and female effects on sperm occupancy, owing to potential covariation between sperm quality and male attractiveness. Specifically, heat-treated male sperm may intrinsically be poor at entering, and persisting in the female storage organs. Conversely, females may be cryptically selecting against such sperm [107], as they are likely to carry damaged genetic material with consequences for offspring fitness. *T. castaneum* females have demonstrable abilities to restrict correct sperm placement [74] and bias paternity shares [107–109] away from unattractive males. Furthermore, such female influence seems to be restricted in scenarios where bursal musculature control is compromised by female death or anaesthetization [74,86,107,108]. Consequently, our observation that sperm from heated males were consistently absent from places like the anterior bursa could indicate active discrimination against them by the females. Moreover, previous data show that females may discriminate against heat-stressed sperm, as they have a greater propensity to seek mating opportunities after being paired with heated males [33]. However, evidence for cryptic choice against heated sperm relative to control sperm may be limited, because we found: (i) no difference in the time they took to enter the spermatheca, (ii) that they were lost from the bursa at a similar rate, and (iii) that they were not selected against in relation to females mated previously.

## 5. Conclusion

In conclusion, sperm produced from heat-stressed males were less successful at occupying the female storage sites advantageous for fertilizations. Our research improves our mechanistic understanding of heat-induced male infertility, which could underlie and help explain climate-related population declines. Here, we tested common scenarios of prolonged sperm storage and sperm competition. Perhaps reassuringly, the losses from thermal stress were only additive, rather than synergistic, supporting the accuracy of previous experimental work where such factors were unaccounted for. We also highlight that future work should consider the potential site-specificity of factors influencing sperm storage in the female reproductive tract.

## Data Availability

The dataset and related R codes are available from the Dryad Digital Repository [110]. Electronic supplementary material is available online [111].

## Dedication

We dedicate this study to our friend, PI and mentor (Prof. Matthew JG Gage; 1967–2022) whose passion, energy and guidance brought us here. This is to those cherishable memories with Matt.
